# Smart Objects for Clinical Rehabilitation and Education: A Scoping Review of Design, Sensing, and Validation

**DOI:** 10.3390/s26185812

**Published:** 2026-09-14

**Authors:** Lorenzo Pugi, Laura Fiorini, Marco Vincenzo Maselli, Filippo Cavallo

**Affiliations:** Department of Industrial Engineering, University of Florence, 50121 Firenze, Italy; lorenzo.pugi@unifi.it (L.P.); marcovincenzo.maselli@unifi.it (M.V.M.); filippo.cavallo@unifi.it (F.C.)

**Keywords:** smart objects, human–object interaction, rehabilitation technologies, motor and cognitive skill assessment, educational tools

## Abstract

Interaction with physical objects plays a key role in the development, assessment, and treatment of cognitive and motor skills across the lifespan. In recent years, this interaction has been enhanced by smart objects, namely everyday items augmented with embedded sensing, processing, and feedback capabilities. These objects enable objective behavioural data collection while preserving natural and engaging human–object interactions. However, the literature remains fragmented and often focused on specific applications or interaction paradigms. This scoping review provides an overview of smart objects developed for clinical and educational contexts, with intended uses including assessment, treatment, training, education, and data collection to support machine learning approaches. Forty-two studies published from 2010 up to the final search date of 29 June 2026 were analysed following the PRISMA-ScR guidelines. The review adopts a design- and hardware-oriented perspective, classifying smart objects according to physical shape, intended use, target population, and validation level. Particular attention is given to embedded electronic components, measured parameters, and the exploitation of sensing and feedback technologies during human–object interaction. By synthesising current solutions, this review highlights emerging trends, recurring limitations, and open challenges related to design choices, technological constraints, and experimental validation, supporting the development of robust, adaptable, and real-world-ready smart objects.

## 1. Introduction

The use of objects is a fundamental aspect of human interaction, providing the primary means through which individuals engage with their environment and carry out Activities of Daily Living (ADL) [1,2]. From early childhood, exploring and manipulating physical objects is crucial in the development of multiple abilities, including cognitive, perceptual, motor, linguistic, and communicative skills [3]. Traditionally, interacting with objects has been leveraged in assessment, treatment, and learning contexts. For instance, standardised developmental tests frequently include activities such as stacking cubes to measure psychomotor development [3]. Similarly, the analysis of complex motor tasks, like handwriting on paper, has served as a valuable biomarker for evaluating cognitive decline or neurological dysfunction [4,5]. Moreover, atypical, repetitive or restricted manipulation behaviours are often associated with developmental motor delays or conditions such as Autism Spectrum Conditions (ASCs) [6].

Despite their widespread use, these methods present several limitations: they often require highly trained specialists and dedicated equipment, involve time-consuming video analysis, rely partly on subjective scoring, and do not provide continuous data during real-life activities. A novel and promising approach to overcome these limitations is the use of sensorised objects, which enable objective measurement of kinematic and physiological parameters during task execution. In addition, their tangible and playful nature can enhance user engagement and motivation, making it possible to support unsupervised or minimally supervised assessment and rehabilitation.

Within this context, smart objects are physical, directly manipulable objects, such as toys, spoons, cubes, and mugs, augmented with sensing and/or active feedback capabilities and associated electronic components, allowing them to acquire digital information about human–object interaction and/or provide interactive feedback [7,8]. By integrating technologies like Inertial Measurement Units (IMUs), force sensors, LEDs, and speakers, they can provide a wide range of interactive experiences while generating digital information about human–object interactions. In recent years, the development of smart objects is focused on healthcare, rehabilitation, and educational contexts, due to their features to transform traditional methods into engaging and quantifiable experiences.

Over the last decade, there were other literature reviews that addressed similar research topics. Molina et al. [9] investigated the key benefits and challenges of adopting tangible user interfaces (TUIs) in programming education. Ref. [10] analysed existing TUIs aimed at enhancing social interaction among older adults and outlined future research directions. Ref. [11] presented an overview of TUI applications across different areas, including medicine and education. Komis et al. [12] categorised smart toys according to their technological and educational affordances, with a specific focus on early childhood and primary education. de Albuquerque & Kelner [13] classified Toy User Interfaces (ToyUI) into four application fields, also considering physical characteristics such as materials, size, shape, and symbolic representation. Overall, these reviews primarily focus on TUIs, which are defined as “*real physical objects that, when manipulated, can control a virtual space and represent digital information.*” [14]. However, they do not systematically examine smart objects from a **design- and hardware-centred perspective**. In fact, existing reviews do not analyse how sensing technologies, embedded electronics, feedback mechanisms, and physical form influence the use of smart objects as autonomous tools for assessment, treatment, education, or data-driven applications. As a result, key challenges related to clinical validation, real-world engagement, long-term use, and integration into everyday workflows remain scattered across individual studies and have not been critically synthesised.

This gap also distinguishes the present review from adjacent but broader reviews on smart-device-based rehabilitation, such as Krumina et al. [15], which focuses on smart device interventions for hand motor recovery after stroke, and from reviews of portable technologies for infant motor assessment, such as Deng et al. [16], which addresses a specific clinical population and assessment context. Similarly, recent reviews on IoT devices in education and wearable sensors for learning analytics, including Tsipianitis et al. [17] and Hong et al. [18], primarily frame these technologies in terms of educational affordances, IoT infrastructures, wearable data streams, or learning analytics challenges, rather than as physically embodied smart objects whose hardware architecture and interaction design directly influence usability, engagement, data quality, and deployment.

To fill these gaps, this scoping review adopts an application-oriented perspective, treating smart objects as physical tools operating in real environments rather than as interfaces to digital systems. The aim of this review is to provide an overview of smart objects developed for the assessment, treatment, and education of cognitive and motor skills. Specifically, this scoping review aims to

i. Classify smart objects according to their design characteristics and intended use;
ii. Analyse the electronic components, sensing, reasoning, and feedback technologies adopted and the measured parameters;
iii. Examine the populations in which these objects have been tested;
iv. Identify existing gaps and methodological limitations so as to define future research directions to support the development of next-generation smart objects for clinical and educational applications.

This review is organised as follows: Section 2 describes the search strategy, selection criteria, and data extraction process; Section 3 presents the main findings; and Section 4 critically analyses current limitations, challenges, and future research opportunities.

## 2. Materials and Methods

### 2.1. Protocol

This scoping review was conducted following the PRISMA-ScR (Preferred Reporting Items for Systematic Reviews and Meta-Analyses extension for scoping review) guidelines [19] and guided by the JBI Manual for Evidence Synthesis. The review protocol was not prospectively registered or published.

### 2.2. Search Strategy

The research was performed on three databases, namely Scopus, Web of Science, and PubMed. Since each database uses different field tags and search syntax, the search strategy was adapted accordingly while maintaining the same combination of search concepts. The exact search strings were as follows:

**Scopus**:

TITLE-ABS-KEY-AUTH (“smart object*” OR “Smart toy*” OR “sensorised object*” OR “tangible user interface” OR “TUI” OR “Sensor-augmented toy*”) AND (“cognitive impairments” OR “fine motor skills” OR “rehabilitation” OR “hand-object interaction” OR “hand rehabilitation” OR “pediatric rehabilitation” OR “motor skill assessment” OR “learning platform” OR “Cognitive assessment” OR “motoric Impairments”).

**Web of Science**:

ALL = ((“smart object*” OR “Smart toy*” OR “sensorised object*” OR “tangible user interface” OR “TUI” OR “Sensor-augmented toy*”) AND (“cognitive impairments” OR “fine motor skills” OR “rehabilitation” OR “hand-object interaction” OR “hand rehabilitation” OR “pediatric rehabilitation” OR “motor skill assessment” OR “learning platform” OR “Cognitive assessment” OR “motoric Impairments”)).

**PubMed**:

((“smart object*”[Title/Abstract]) OR (“Smart toy*”[Title/Abstract]) OR (“sensorised object*”[Title/Abstract]) OR (“tangible user interface”[Title/Abstract]) OR (“Sensor-augmented toy*”[Title/Abstract])) AND ((“cognitive impairments”) OR (“fine motor skills”) OR (“rehabilitation”) OR (“hand-object interaction”) OR (“hand rehabilitation”) OR (“pediatric rehabilitation”) OR (“motor skill assessment”) OR (“learning platform”) OR (“Cognitive assessment”) OR (“motoric Impairments”)).

The search included studies published in English between 1 January 2010 and the final search date of 29 June 2026.

### 2.3. Selection Criteria

For the purpose of this review, a smart object was defined as a physical and directly manipulable object incorporating, or physically integrated with, sensing and/or active feedback components. These components enable the object itself to acquire information about human–object interaction or provide interactive feedback. Wearable devices in which sensing components were worn on the user’s body, environmental sensing systems, and rehabilitation equipment in which sensing was external to the manipulated object were not considered smart objects. Tangible user interfaces and sensor-augmented conventional objects were included only when they met this operational definition.

As a first screening step, all duplicate papers were discarded manually by comparing the title, authors, and publication year into a single Microsoft Excel spreadsheet. Secondly, the titles and abstracts of the retrieved papers were examined according to the exclusion criteria set by one of the reviewers. In particular, the exclusion criteria used in this review were as follows: (1) any smart object was not mentioned in the work; (2) the paper presented an object that could not be handled (i.e., smart room, smart environment, or AR circuit constructor); (3) the object was equipped only with an RFID tag or QR code, or it did not incorporate any sensing component or active feedback element; (4) the work presents a lack of information about the types of electronic components that the objects were equipped with (i.e., accelerometer, gyroscope, force sensors, or/and physiological sensors); (5) the paper was not submitted to a journal or a conference published in the last sixteen years; (6) the paper was not written in English; (7) the paper was an abstract, a review article, or a chapter published in a non-scientific book; (8) the article was not fully accessible; and (9) papers written by the same authors describing the same object were excluded, and only the most recent version was included. The reports selected for full-text assessment were divided between two reviewers, who assessed eligibility and extracted the relevant data. Whenever uncertainties regarding eligibility or data extraction arose, the report was discussed by both reviewers until consensus was reached.

## 3. Results

### 3.1. Overview

**Figure 1:**
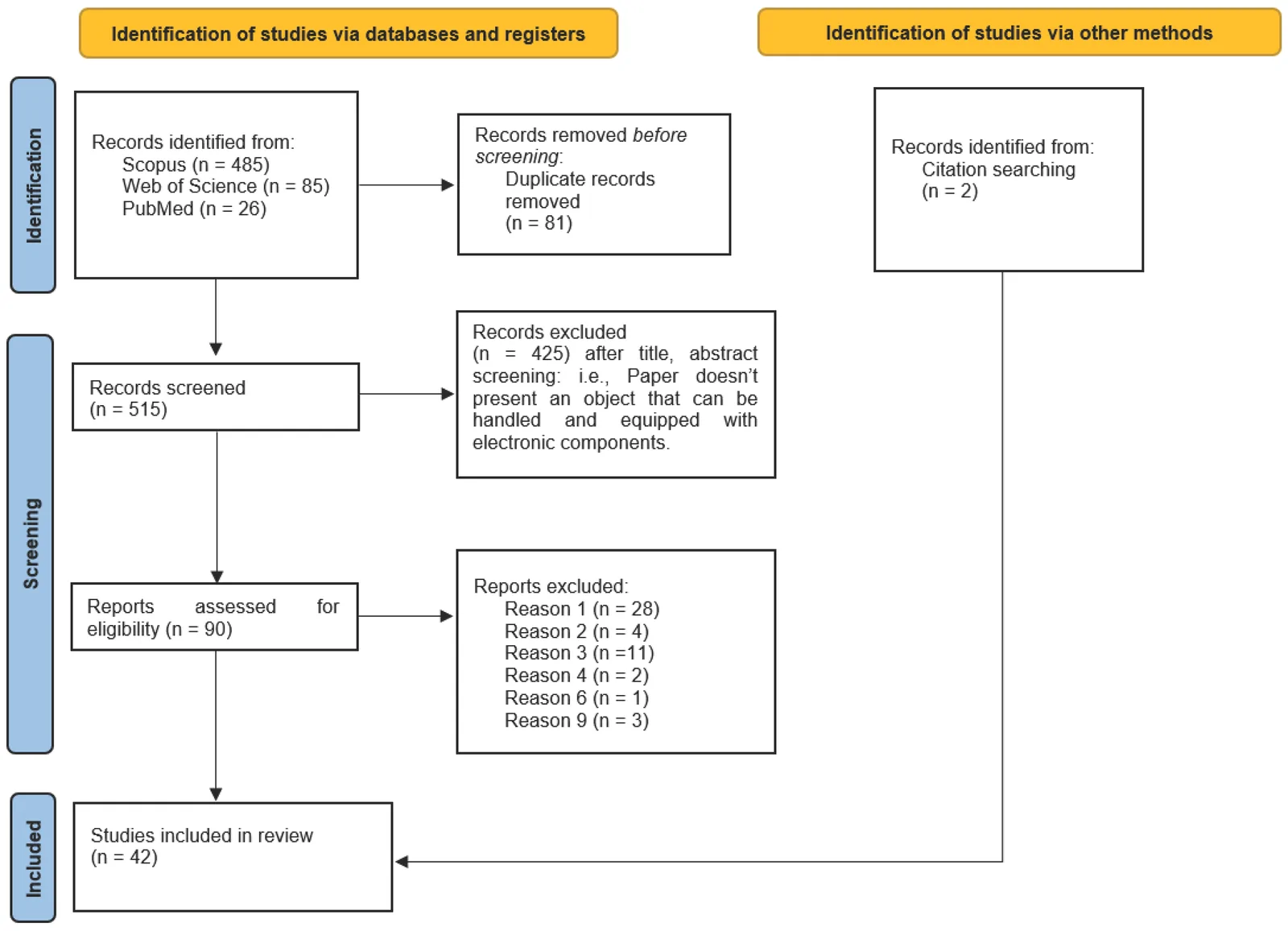
PRISMA flow diagram illustrating the study selection process.

A total of 596 records were retrieved from Scopus (*n* = 485), PubMed (*n* = 26), and Web of Science (*n* = 85). After removing 81 duplicates, 515 records underwent title/abstract screening, of which 425 were excluded. Ninety reports were therefore sought for retrieval; 1 could not be retrieved, leaving 89 reports for full-text eligibility assessment. Following full-text assessment, 49 reports were excluded, and 40 studies identified through the database search were included. In addition, two studies already known to the authors, but not retrieved by the database searches, were assessed according to the same eligibility criteria and included, resulting in a final total of 42 studies. The number of studies at each stage of the process is presented in accordance with the PRISMA (Preferred Reporting Items for Systematic Reviews and Meta-Analyses) flow diagram, as shown Figure 1.

**Figure 2:**
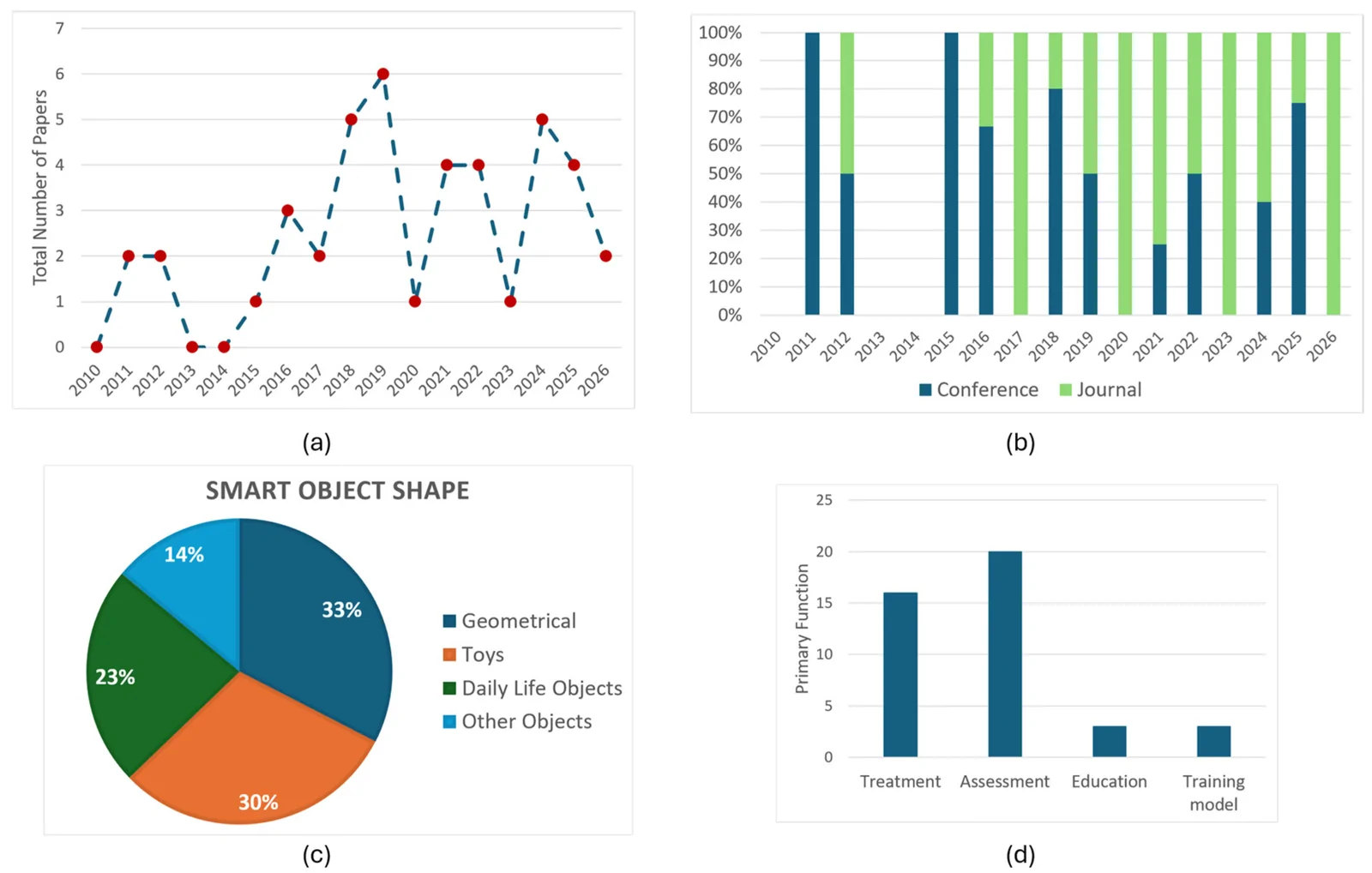
Overview of the analysed studies and smart objects: (**a**) distribution of publications per year (2010–2026); (**b**) distribution of source type (journal vs. conference papers); (**c**) classification of smart objects according to physical shape; (**d**) distribution of smart objects according to intended use.

Over the past few years, there has been a growing interest in the design and development of smart objects aimed at assessing or treating patients, as reflected in Figure 2a, displaying the distribution of the selected papers from 2010 to 2026. Most of the papers were published from 2011, with a peak in 2018 and 2019. However, from 2020 to 2024, the number of publications decreased. Figure 2b describes the distribution of the analysed papers according to the source type (i.e., conference or journal paper).

The authors classified the smart objects used in analysed studies based on the following criteria: design (namely cross-object and specific design), the ultimate intended use of the smart object in the studies where they were involved (e.g., treatment or assessment; Figure 2d), and the *human skill* target (e.g., motor or cognitive).

Additionally, the types of electronic components, the measured parameters, and whether the studies involved target-user or non-target-user evaluation were also analysed. Specifically, the studies that presented multiple objects with varying shapes but equipped with the same electronic module were classified as *cross-object design*. Conversely, studies that developed one or more objects with electronic components selected to their specific shape were classified as *object-specific design*. Across the 42 included studies, 49 smart objects were identified: 43 object-specific objects and 6 cross-object systems. In cross-object systems, the same electronic module was embedded in multiple physical forms; therefore, each system was counted as a single smart object regardless of the number of shapes in which the module was used.

### 3.2. Intended Use of Smart Objects

In this section, the authors discuss the purpose of the objects (Figure 2d), classifying them according to their intended use using the following definitions:

- **Assessment**: All smart objects in this category are developed in order to collect, analyse, and interpret user-related data. For example, these objects could be used to evaluate cognitive or motor performance during activities, to enrich the decision making of clinicians, or to support clinicians in the assessment. Moreover, their design can be inspired by traditional tools used in standardised tests or follow the same assessment protocols, thus enabling the digitalisation and enhancement of these evaluations.
- **Treatment**: The smart objects in this category are designed to either facilitate rehabilitation activities, such as hand rehabilitation or post-stroke therapy, or to function as coaching tools by monitoring specific cognitive or motor functions during the treatment. Similarly, their design can be inspired by conventional therapeutic tools or replicate the same intervention protocols, allowing the digitalisation of traditional treatment practices.
- **Education**: These smart objects are developed for educational purpose, such as teaching mathematics and geometry, or for enhancing cognitive skills.
- **Training**: The authors refer to the objects designed or employed to gather data that are meant to be used for training neural network/machine learning (ML) models for activity recognition tasks.

Additionally, the analysed smart objects were also categorised, inside each group, according to the human targeted skill, such as health status, cognitive ability, motor skills, or activity recognition, as reported in Table 1.

#### 3.2.1. Smart Objects for Assessments

Among the studies involved in this work, six of them developed smart objects for cognitive assessment. For instance, Crovari et al. [20] designed and developed SAM, a dolphin-shaped smart toy, to engage children with Neurodevelopmental Disorders (NDDs) in a variety of tasks. Lee et al. [21] developed Sensor-Integrated Geometric Blocks (SIG-Blocks) to be used in tangible Geometric Games (TAG-Games) for automated cognitive assessment. Their study investigated whether performance in TAG-Games was correlated with scores obtained from three subtests of the Wechsler Adult Intelligence Scale—Fourth Edition (WAIS–IV): Block Design (BD), Matrix Reasoning (MR), and Digit Span (DS). To collect information about the duration and the modality of the interaction of playing with, Westeyn et al. [22] presented a set of smart toys that can be used to characterise objectively how children play.

On the other hand, smart objects that were designed to perform motor assessment have been developed in 11 studies. For instance, sensorised tokens were used in a board game called getThatCheese in order to assess fine motor skills in children [23]. As another example, Lunardini et al. [24] equipped an ink pen with force and motions sensors for quantify and assess, in an ecological way, daily life handwriting. Brons et al. [25] used sensor-augmented toys to predict the fine M-ABC outcomes with the aim of assessing children’s fine motor skills. One study designed three cubes embedded with physiological sensors to measure children’s physiological parameters to assess their health [26]. Moreover, Leo et al. [27] and Biswas et al. [28] assessed both motor and cognitive performance. The former developed a cube, named iCube, to investigate how spatial skills influence performance in a haptic task, whereas the latter created a set of smart cubes aimed at monitoring, characterising, and enhancing human spatial reasoning.

#### 3.2.2. Smart Objects for Treatment Purposes

Smart objects for motor treatment sessions were developed in 12 studies. An example is presented in [29], where an electronic dumbbell was developed to help patients to train their injured wrists at their home. The object, equipped with an accelerometer and a vibrotactile actuator, was used as a gamer control for the ping-pong game. Stephens-Fripp et al. [30] developed a prototype of a sensorised object designed to measure, record, and transmit the gripping force of prosthetic hands. In the future, this device could be used to provide sensory feedback, helping users to better control their grasping force. To monitor motor functions of stroke patients during rehabilitation sessions, Bobin et al. [31] created an ecosystem of smart objects, inspired by the Action Research Arm Test (ARAT) protocol and the conventional rehabilitation tools. Within cognitive treatment, two studies introduced smart toys: one supporting emotion regulation therapy [32] and the other enhancing engagement in children with difficulties in interacting with their environment [33].

Two studies presented smart objects addressing both motor and cognitive treatment: Solorzano Alcivar et al. [34] designed and created a stackable toy to enhance children’s creativity, hand–eye coordination, and fine motor skills, while Vondrak et al. [35] created a set of smart cubes and an interactive game mat for neurocognitive rehabilitation, stimulating memory, attention, and executive function.

#### 3.2.3. Smart Objects for Educational Purposes

Three studies developed smart objects designed to help children in learning school subjects. An example of a smart object used as a tool for teaching geometry is presented by Dujić Rodić et al. [36]. They integrated an LCD screen and InfraRed sensor into a plush giraffe toy, enabling it to recognise the hand movements of children and correct them using a machine learning system. Moreover, Cholitha Hettiarachchi et al. [37] proposed a novel tangible interface called Tangicubes. TangiGuru is an e-learning platform that consists of 12 Tangicubes. TangiCubes are equipped with an OLED display, LEDs, vibration motor, and sensors, designed for developing cognitive skills in children and to help them learn better when playing and exploring the physical world.

#### 3.2.4. Smart Objects for Training AI Models

Three studies presented smart objects designed to collect data for training AI models. For example, Patel et al. [38] evaluated the performance of their classifier algorithm in identifying manual exploratory behaviours based on the sensor data. Bartolomei et al. [39] reported the accuracy of a model trained with data gathered through their smart object to classify user actions associated with symbolic play, enabling the system to provide appropriate feedback to encourage symbolic play in children with autism spectrum conditions (ASCs).

#### 3.2.5. Domain Applications

Currently, these smart objects are used for a wide range of functions that cluster into several domain applications. A first domain concerns *Motor Rehabilitation and Physical Therapy* [24,29,30,31,40,41,42,43,44,45,46,47], where smart objects act as tools for structured motor training or for upper-limb recovery of post-stroke patients or those with wrist or hand injuries. Other objects serve the functional purpose of supporting *Fine Motor Skills*, *Visual–Motor Integration*, *and Cognitive–Motor Assessment* [21,23,25,27,28,38,48], becoming an accessible tool for quantifying a user’s motor and cognitive skills. Additionally, some smart objects are employed for *Neurodevelopmental Assessment and Early Detection* [3,22,49,50,51,52], where sensorised toys capture atypical behavioural patterns during play to support the diagnosis of neurodevelopmental impairments or autism in children. Complementarily, there are smart objects designed for *Cognitive*, *Emotional*, *and Social Development* [20,32,33,34,35,37,39,53], leveraging interactive or affective feedback to promote engagement or socio-emotional skills. Beyond clinical aims, smart objects have also been explored for *Activity Cueing*, *Coaching*, *and Human–Object Interaction* [1,54,55,56], demonstrating how embedding actuation and sensing into everyday items can support the sequencing of daily actions or reduce cognitive load. They have also been employed in *Educational and STEM-Based Learning* [36,57,58] environments as well as *Health Monitoring and Physiological Tracking* [26,59], integrating embedded sensing to capture physiological or behavioural indicators.

### 3.3. The Shape of the Smart Object

Concerning smart objects with an object-specific design, the authors categorised the shapes of smart objects as geometrical shapes, toys, daily life objects, and other objects, as shown in Figure 2c. Geometrical shapes, such as cubes, spheres, and parallelepipeds, are used widely as smart object shapes (14/43, 33%). These types of shapes are mainly used in game activities where it is required to build something with cubes [3], to recreate a displayed image using objects [21], or to control a virtual game [56]. Many studies endowed daily life objects (10/43, 23%), such as a cup, mug, jug, or spoon, equipped with electronic components. These smart objects were designed for various purposes, including assessing motor skills [52], recognising human activity, and identifying individuals [55], or to monitor post-stroke rehabilitation during daily activities at home [40]. To monitor, assess, and teach children, many studies incorporated sensors and other electronic components inside toys (13/43, 30%) such as plush toys, toy cars, toy planes, and LEGO. For example, Lanini et al. [50] equipped a truck and a plane with sensors to detect the movements and orientation of the toy in real time while it is being played with. Similarly, Udayagiri et al. [51] created a panda head with a Lossy Force Sensor to acquire data on the interactions of infants.

Finally, a few studies (6/43, 14%) incorporated electronic components into objects such as crutches, used to measure physiological parameters in physiotherapy [45]; dumbbells, designed for wrist rehabilitation [29]; or tokens, used in a board game for fine motor skills assessment [23]. Regarding smart objects with a cross-object Design, as described in Section 3.1, there is not a predefined shape. Indeed, the same electronic module was embedded inside in various types of objects, ranging from commercial to manufactured objects, such as soft toys, printed cubes, spoons, and balls. For example, in the work of Moraiti et al. [48] occupational therapists created custom smart objects using a toolkit provided by authors. The Table 1 and Table S1 summarise the types of shapes used across the studies included in this review.

### 3.4. Electronic Components

All the smart objects discussed in the 42 studies reviewed were equipped with various electronic components, as reported in Table 1 and Table S1. For simplicity, the authors categorised these components into four groups: sensing, feedback, communication/identification, and reasoning elements. Sensing elements collect data during human–object interaction, while feedback elements provide visual, auditory, or vibrotactile cues. Communication/identification elements support either wireless data transmission or the identification of nearby objects, whereas reasoning elements process sensor data, control feedback components, and extract relevant features. Nineteen studies integrated Sensing and reasoning elements into their objects, while 21 studies also integrated feedback elements. Only 2 studies embedded reasoning and feedback elements into smart objects.

Most of the smart objects have as **reasoning element** a Microcontroller. Only a few objects (4 out of 49) did not include an embedded microcontroller and instead transmitted data directly to a PC or external receiver for processing and feature extraction.

**Communication/identification elements** included technologies used either for wireless data transmission or for object identification. Wi-Fi and Bluetooth/BLE were the most commonly adopted communication technologies, being integrated into 15 and 12 of the 49 smart objects, respectively. In addition, five smart objects incorporated RFID and/or NFC technologies, primarily for identifying or recognising nearby objects or tags rather than for continuous data transmission; these five smart objects also include Wi-Fi or Bluetooth/BLE connectivity. For 22 out of 49 smart objects, no dedicated wireless transmission module was reported, or this information was not specified. **The sensing elements** have been further divided into subgroups according to the measured event: inertial sensors, optical sensors, force sensors, physiological sensors, tactile sensors, environment sensors, and tag sensors. Inertial sensors are devices that measure the motion and orientation of an object using its inertia. These sensors, such as acceleration and/or angular velocity and/or magnetometers, are employed to measure the dynamic of smart objects. Some objects are equipped with only accelerometer (7 out of 49) or gyroscope (4 out of 49), rather than an Inertial Measurement Unit (IMU) with 6 Degrees of Freedom (6-DoF; 6 out of 49) or IMU 9-DoF (17 out of 49). Optical sensors are devices that detect light and convert it into an electrical signal, such as InfraRed (IR), optical fibres or Light Dependent Resistor (LDR). These sensors can be applied to measure a force, to detect another close object, or whether an object is placed on top of another one. A total of 9 Smart objects are equipped with this group of sensors. The force sensors measure the force applied to it and convert that force into an electrical signal. This signal can then be measured, converted, and standardised to indicate the magnitude of the applied force to an object. Some smart objects are embedded with force sensors like Force Sensing Resistor (FSR), load cell, strain gauge, pressure sensor or Force-sensing Linear Potentiometers (FSLP). Physiological sensors are a type of sensor that measure signals generated by the human body, such as heart rate, blood pressure, and respiration. The physiological sensor employed are Pulse/Oxygen Saturation Sensor, body temperature sensors, differential pressure sensor and photoplethysmography (PPG). Tactile sensors are devices that detect physical contact, providing information about pressure, force and texture. These kinds of sensors are embedded in few smart objects, and include capacitive sensors, capacitive button, and touch and pulse sensor (TAPS). Environment sensors measure and monitor various parameters of the surrounding environment. Six out of 49 objects are equipped with these sensors, such as barometer, humidity sensors, thermometer and liquid level detection. Finally, tag sensors leverage wireless technologies, such as Radio Frequency Identification (RFID) and Near Field Communication (NFC), in order to enable data transmission. These sensors are attached to objects to identify and track them.

**Figure 3:**
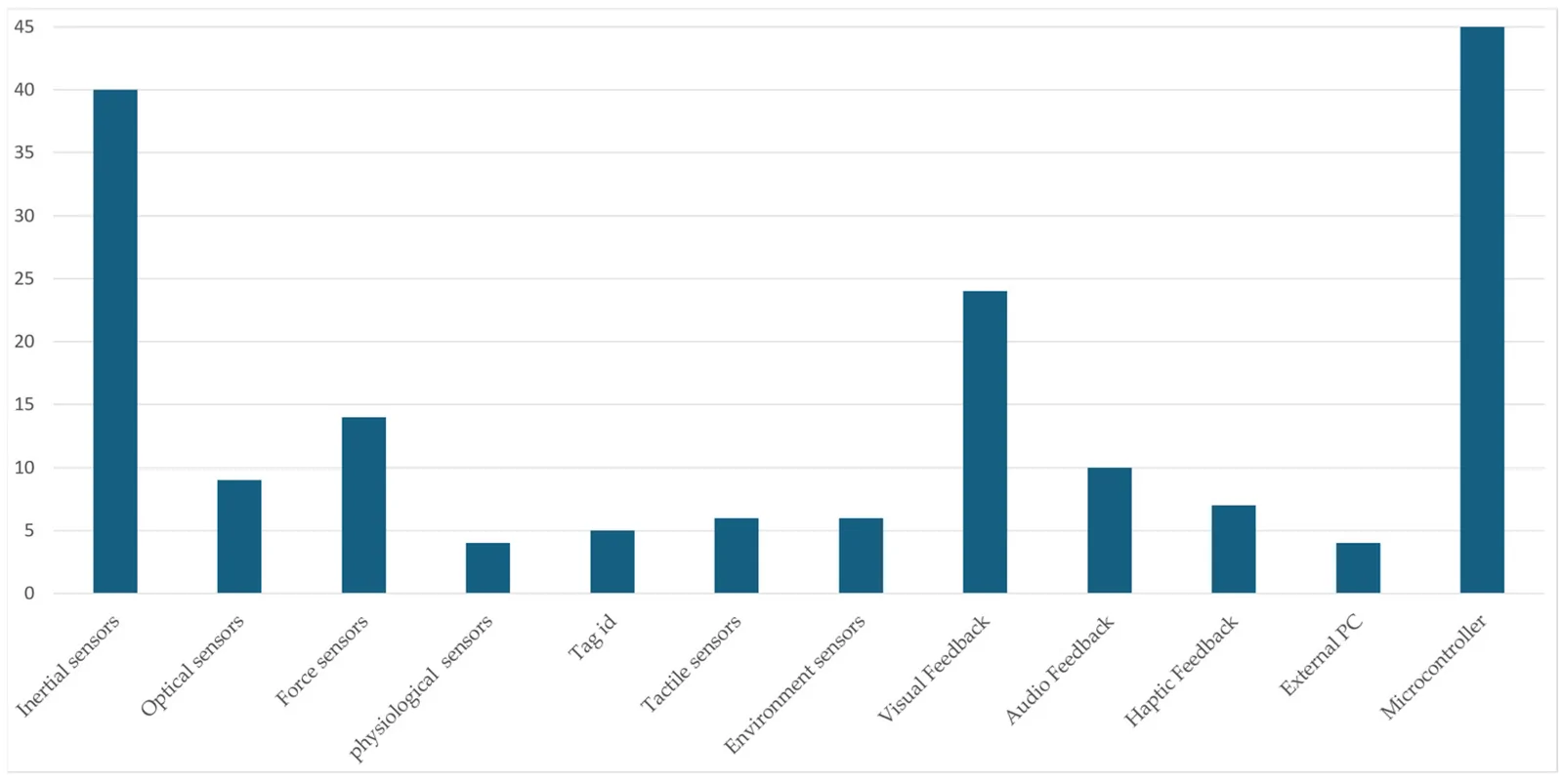
Distribution of electronic components integrated into the analysed smart objects.

The **feedback elements** are devices that provide visual, auditory, and haptic feedback through components such as LEDs, buzzers, and vibration motors. LEDs (20 out of 49) are the most used electronic components for visual feedback. Three smart objects were equipped with display capable of showing figures, geometric shapes, or similar visual content. For auditory and haptic feedback, smart objects were typically equipped with buzzers (10 out of 49) and vibration motors (7 out of 49). The Figure 3 shows the distribution of these elements into smart objects.

### 3.5. Measured Parameters

As outlined in the “*Electronic components*” section, the choice of sensors embedded in each object was driven by the parameters to be measured. In this context, it is worth noting that the majority of the smart objects included in the analysis were designed to capture manipulation parameters (Table 1).

Most studies focus on kinematic parameters, mainly 3D acceleration, rotation, and orientation, which enable the detection of how the object is manipulated and whether tremors occur during movement. These signals are often processed to derive or extract features such as velocity, jerk, object direction, and inclination, providing a more detailed representation of motor behaviour.

Additionally, force parameters are used to assess grasp strength and to detect lifting action. In some cases, force sensors are employed to estimate object deformation and finger positioning, offering insight into grasp strategies and fine motor control.

Finally, a smaller subset of studies extends beyond kinematic and force measures to include physiological signals and task-specific parameters, such as hand gesture recognition or environmental variables (e.g., water level).

**Table 1:** Overview of smart objects according to intended use, target skills, hardware configuration, and measured parameters.

Reference	Intended Use|Human Target Skills	Number of Objects Used	Shape	Electrical Components	Measured Parameters
[40]	Treatment|Motor	1	Daily life objects|Cup	FSR, IMU 9-DoF, NFC, LED, liquid level detection, MCU WI-FI	Grasping force, 3D acceleration, rotation (tremor detection), level of water inside the object
[1,54]	Treatment|Motor	3	Daily life objects|jug, Cup, Spoon	**jug**: LED, FSR, MCU WI-FI; accelerometer, tilt sensor, speaker **spoon**: accelerometer, vibration motor, LED strip, MCU WI-FI. **cups**: LED, MCU WI-FI	3D Acceleration, object’s inclination and when it has been picked up
[31]	Treatment|Motor	2	Geometrical|Cube, rounded parallelepiped	**Jack**: MCU WI-FI, IMU 9-DoF, FSLP **Cube**: FSR. MCU BLE, IMU 9-DoF	3D acceleration, orientation and rotation, and the pressure applied and the fingers position
[44]	Treatment|Motor	2	Toy|LEGO cottage, Crocodile head shape	**LEGO**: load cell, MCU BLE **Crocodile**: capacitive button, MCU BLE	Grasping force and number of pinches
[25]	Assessment|Motor	1	Geometrical|Cube	IMU 6-DoF, MCU BLE, LED	3D acceleration and rotation (Jerk derived from angular velocity)
[57]	Education|Cognitive	1	Toy|Bear	MCU, gyroscope, LED, RFID	3D Orientation and Card validation
[41]	Assessment|Motor	1	Daily life objects|Cup	MCU WI-FI, IMU 9-DoF, Buzzer, LED, Liquid level detection, Strain gauge	3D acceleration, orientation and rotation, level of water inside the object and grasping Force and hand movements
[37]	Education|Cognitive	1	Geometrical|Cube	MCU WI-FI, IMU 6-DoF, LED, IR, display OLED, Vibration motor	3D acceleration and orientation, Close object detection
[32]	Treatment|Cognitive	1	Toy|Plush	MCU, Capacitive touch, Gyroscope, Vibration motor, FSR	3D rotation, Grasping Force and Touch detection
[20]	Assessment|Cognitive	1	Toy|Dolphin	MCU WI-FI, RFID, LED, Buzzer, IMU 6-DoF, Tactile sensor	3D acceleration and rotation, Card identification, Touch detection
[36]	Education|Cognitive	1	Toy|Giraffe	MCU, IR, Display LCD, Buzzer	Hand detection
[58]	Assessment|Cognitive	9	Geometrical|Cube	MCU WI-FI, RFID, LED, Buzzer, IMU 9-DoF	3D acceleration, orientation and rotation, Object Identification
[59]	Assessment|Motor	1	Daily life objects|Water bottle	MCU WI-FI, TAPS, PPG, IMU 6-DoF,	3D acceleration and rotation, Heart rate Variability and Touch detection
[49]	Assessment|Motor	5	Cross-object Design|Doll, spoon, toy car, cube and ball	MCU BLE, IMU 9-DoF, Barometer, Thermometer and Humidity sensor	3D acceleration, orientation and rotation
[3]	Assessment|Motor	4	Geometrical|Cube	MCU, IMU 9-DoF, LDR, tilt sensor, LED, Buzzer	3D acceleration, orientation and rotation, (max and min acceleration, number of movement)
[42]	Treatment|Motor	1	Other objects|Stress ball	MCU, FSR, Accelerometer, Vibration motor	3D acceleration, Grasping Force
[29]	Treatment|Motor	1	Other objects|Dumbbell	MCU, Vibration motor, Accelerometer	3D acceleration (detect pronation/supination range of motions)
[50]	Assessment|Cognitive	2	Toy|Track, Plane	**Track:** IMU 9-DoF, MCU WI-FI **Plane:** IMU 9-DoF, MCU WI-FI	3D acceleration, orientation and rotation
[21]	Assessment|Cognitive	4	Geometrical|Cube	Accelerometer, MCU, reflective optical sensor	3D acceleration, Close object detection
[27]	Assessment|Motor/Cognitive	1	Geometrical|Cube	IMU 9-DoF, Capacitive Button, motion processing unit (MPU)	3D acceleration, orientation and rotation, Touch detection
[24]	Assessment|Motor	1	Daily life object|Ink	MCU BLE, LED, load cell, IMU 6-DoF	3D acceleration and rotation (Tremor detection), Writing Force (mean writing force)
[45]	Assessment|Motor	1	Other objects|Crutch	FSR, IMU 9-DoF, MCU BLE	Grasping Force and object inclination
[43]	Treatment|Motor	1	Cross-object Design|ball	IMU 9-DoF, External receiver	3D acceleration and rotation
[23]	Assessment|Motor	1	Other objects|Token	MCU, Accelerometer, LED	3D acceleration (Jerk derived from acceleration)
[48]	Treatment|Motor	8	Cross-object Design|floor mat, cross-controller, polar bear, pillow, gloves, ghost, duck, socks	MCU, pressure sensor	Deformation of objects
[55]	Training Model|Activity recognition	1	Daily life object|Coaster of a jug	IMU 9-DoF, FSR, MCU WI-FI	3D acceleration, orientation and rotation, lifting Force
[52]	Assessment|Motor	4	Daily life object|Spoon, Marker, Toy|Car Toy, Rattle toy	**spoon**: MCU, gyroscope **marker**: MCU, gyroscope **car toy**: rotatory encoder, MCU, **Rattle toy**: IMU 6-DoF, MCU	3D acceleration and rotation, Number of wheel rotations (Object direction)
[30]	Treatment|Motor	1	Geometrical|Cube	FSR, MCU BLE	Grasping Force
[51]	Assessment|Motor	1	Toy|Panda head	Optical fibers, PC	Loss of emitted light (Grasping Force derived)
[56]	Assessment|Motor	1	Geometrical|Spherical	MCU, IMU 9-DoF, barometer, vibration motor, Light Dependent Resistor	3D acceleration, orientation and rotation, Altitude, Hand proximity
[26]	Assessment|Health monitoring	3	Geometrical| Cube	MCU WI-FI, Pulse/Oxygen Saturation Sensor, Thermometer, differential pressure Sensor, Buzzer	Heart rate variability, Blood oxygen saturation, body temperature, lung volume, skin conductance
[53]	Assessment|Cognitive	3	Cross-object Design|Toy Blocks	IMU 9-DoF, MCU WI-FI	3D acceleration, orientation and rotation (holding time, moving time, standing time)
[22]	Assessment|Cognitive	7	Cross-object Design|Ring, blocks, Caterpillar, Domes, LEGO, Lid, Puppy	Capacitive touch, Accelerometer, MCU	3D Acceleration
[46]	Treatment|Motor	1	Geometrical|Cylinder	MCU, pressure sensor	Finger Force
[38]	Training model|Activity recognition	5	Cross-object Design|ball, cube	PC, IMU 9-DoF	3D acceleration, orientation and rotation
[39]	Training model|Activity recognition	1	Other Objects|Shape not defined	MCU BLE, LED, IMU 9-DoF, Buzzer	3D acceleration, orientation and rotation.
[34]	Treatment|Motor/Cognitive	1	Toy|Duck	MCU, LED, buzzer	No sensor-derived parameter reported
[35]	Treatment|Motor/Cognitive	6	Geometrical|Cube	MCU BLE, LED, IR, Buzzer	Object detection
[33]	Treatment|Cognitive	1	Other Objects|Plastic box	MCU BLE, LED, IR, Buzzer, IMU 6-DoF, Gesture sensor	3D acceleration and rotation, Hand detection and gesture
[47]	Treatment|Motor	1	Toy|Monster toy	MCU WI-FI, IMU 6-DoF, display OLED	3D acceleration and rotation
[28]	Assessment|Motor/Cognitive	5	Geometrical|Cube	MCU, LED, buzzer, IMU 6-DoF, vibration motor, near-field magnetic induction (NFMI)	3D acceleration and rotation, Object Identification

### 3.6. Experimental Evaluation and Target-Population Involvement

The 42 studies included in this review were divided into two categories according to the involvement of the intended target population: **Non-target-user evaluation** (14 out of 42) and **Target-user evaluation** (28 out of 42). Non-target-user evaluation included studies in which the intended target population was not involved, either because the smart object was preliminarily tested with non-target participants or because no user testing was conducted. In contrast, Target-user evaluation included studies in which the smart object was evaluated with the intended target population within experimental, clinical, educational, or real-world protocols to assess its feasibility, usability, performance, or suitability for the intended application.

Among the studies classified as Non-target-user evaluation, several conducted preliminary experiments with non-target participants to assess the accuracy of trained models [38,39,40,51], or to validate technical aspects of the smart objects, such as electronic modules, signal processing, or output signals [1,29,30,42,45,54]. For example, a set of sensorised objects was designed by Baber et al. [54] to support activities of daily life for patients with neurological deficit. In this study, 23 healthy young adults, mean age of 25 years, were recruited at the school of Electronic, Electrical and Systems Engineering, to validate the set of smart objects. The participants carried out three trials: pick-up and pour actions guided by visual and auditory cues, lifting a jug to test whether animation and handle orientation cued the action, and performing logical and illogical tea-making sequences based solely on object cues. The results showed that sounds and object animations were more effective cues than lights alone, and illogical action sequences led to more memory errors than logical ones. Moreover, the authors suggest that such animate, sensorised objects could support stroke and neurocognitive patients in daily activities, though further clinical validation is needed. Instead, six studies only described the smart objects, specifying what they should measure or the types of activities they should support, without conducting any test [33,35,46,59]. Martinho et al. [46] described the design, the fabrication, and the characterisation of pneumatic sensing chambers (PSCs) for a hand rehabilitation device aimed at assessing grasping force in post-stroke patients. No participants were recruited for testing, but instead, mechanical experiments and finite element analysis (FEA) were conducted to compare PSC designs with different geometries in terms of sensitivity, linearity, repeatability, hysteresis and durability. Results indicated that the small half-sphere design was the most suitable option for device development.

For studies classified as Target-user evaluation, the target populations were also categorised by age as follows: children (0–12 years), adolescents (13–19 years), young adults (20–35 years), middle-aged adults (36–55 years), and older adults (56 years and above). The number, age, and health or clinical status of the participants, together with the main characteristics of the experimental evaluation, are summarised in Table S2.

#### 3.6.1. Smart Objects Used with Children

The majority of studies (15 out of 42) focused their effort on the development of smart objects for healthy children [3,22,26,34,36,37,44,49] or for children with impairments in fine motor skills development [23,25], intellectual disability [58], post trauma stress [53], hearing impairments [57] or autism [50,52]. Gutiérrez García et al. [3] validated smart cubes as toy for enhancing decision-making process recruiting 65 (32 males and 33 females) children from 2 to 3 years old. In this work, children were asked to build a tower. The results of these experiments showed that the authors have detected with smart object at least three factors of interest to describe the level of psychomotor development of children: trembling, speed, and accuracy. Brons et al. [25] used a commercial toy, named Futurecube, to classify the motor skill levels of 95 children. The participants, aged 6 to 9 years, included 49 children with fine motor skill difficulties and 46 without any difficulties. The study was conducted in an elementary school, where children played two games, the Roadrunner Game, in which players rotated a cube to keep a spot on its top surface, and the Maze Game, where children moved a white dot through a maze of green dots. The study compared different classifiers and games, evaluating both gameplay features and sensor data. The best-performing classifier achieved an accuracy of 0.76 and an F1 score of 0.70 in predicting the MABC-2 (Movement Assessment Battery for Children—Second Edition), demonstrating the potential of sensor-augmented toys for assessing children’s motor skills. Faraci et al. [49] proposed an innovative set of sensorised toys to facilitate early diagnosis of autism spectrum disorders. They conducted a small pilot study involving children aged between 9 and 15 months. The experimental protocol consisted of the child playing freely with these toys, while certain actions were measured and subsequently identified. Data collected from the sensors embedded inside toys were analysed, and features were extracted for activity detection. Even though the full classification model was not trained due to limited data availability, the study demonstrated the feasibility of distinguishing different types of toy movements through feature extraction and preliminary analysis.

#### 3.6.2. Smart Objects Used with Young Adults

Three studies focused on developing smart objects for healthy young adults to assess cognitive–motor skills [21], to identify the action [55], or to investigate the spatial skills [27]. Peng et al. [55] evaluated a sensorised coaster on a cohort of 26 students in their early 20s to train and assess the model for action recognition. The participants were asked to perform actions with a jug in both a controlled and natural manner. The results indicated low error rates in the controlled scenario, with values of 0.15% when the subjects in the training and testing sets matched and 1.65% when they did not. However, performance decreased in the naturalistic scenario, where the error rates increased to 6.35% and 15.33%, respectively. In addition, the authors developed an i-vector-based approach for subject identification, which achieved an accuracy exceeding 68% in the naturalistic scenario.

#### 3.6.3. Smart Objects Tested with Adults

Varesano & Vernero [56] developed a smart object aimed at being used in video games and leisure activities, which they tested with healthy older adults. Fourteen participants, aged 58 to 84 years, were recruited from a training centre class. Each participant played *Pandagolf*, a game in which the goal was to avoid obstacles with a virtual ball by manipulating the smart object. At the end of the game, participants completed a questionnaire to assess their gaming experience with the smart object. The results indicated that the object was positively evaluated in terms of acceptability.

#### 3.6.4. Smart Objects Used with Multiple Age Groups

Six studies tested their smart objects on participants covering multiple age groups as described above, such as healthy young, middle-aged, and older adults [24]; children and adults [28]; young and older adults’ post-stroke [31]; adolescents, young adults, and middle-aged adults with eating disorders [32]; children and adolescents with cerebral palsy [43]; and adolescents and young adults affected by Neurodevelopmental Disorders (NDDs) [20]. For instance, Meriggi et al. [43] recruited 10 (7 males and 3 females) children and adolescents with cerebral palsy aged 3 to 16 years to test an innovative paediatric rehabilitation protocol based on smart objects. The rehabilitation activities took place in the CARE lab, in which each child was asked to perform three sessions of six exercises with a sensorised ball, such as rolling, transferring from one basket to another, launching, and other activities. The results showed that the smart object can detect differences in movements performed by normal and palsy arms.

## 4. Discussion

This review aimed to provide an overview of the objects equipped with electronic components, called smart objects, designed for different intended uses ranging from rehabilitation and clinical assessment to education and training on motor and cognitive skills. The reviewed literature provides several challenges, reflecting both methodological and technological limitations that still constrain the adoption of smart objects for long-term clinical or educational applications. At the same time, the conducted analysis also highlighted recommendations and research trends that should be carried out in the forthcoming years. The next paragraphs are divided according to the main topics and summarise the identified trends. Finally, Table 2 reports the research guidelines for developing the smart object of the future.

### 4.1. Design and Hardware Selection

The analysed papers show that there is a predominance of object-specific designs, often with geometric shapes, toys, and daily life objects, suggesting that most research has focused on creating smart objects with a fixed and well-defined shape. However, compared with cross-object designs, they may show a lower adaptability to different contexts or user groups. Instead, the cross-object designs are characterised by modular architectures that allow the same electronic module to be embedded in various forms, making them potentially more flexible and reusable across multiple applications. In this sense, future works should be focused on designing modular objects so that the same object can be easily applied to different applications.

The findings of this review indicate that the shape of smart objects reflects the intended use and target population. Indeed, geometrical shapes and toys are mainly adopted in studies involving children, where the playful component supports motivation and exploration. On the other hand, daily life objects such as cups, spoons, or pens are preferred in rehabilitation and assessment with adults, for their capability to enable the measurement of gestures that are ecologically valid and meaningful in everyday life. These findings highlight that when developing a smart object, it is essential to consider not only the electronic components but also its shape in order to ensure a familiar and a natural interaction with the user. In particular, the objects should have three main characteristics, which are attractiveness for capturing interest, solid materials for damage prevention, and shape, size, and ergonomics features suitable for the target users. For example, Borghese et al. [44] affirmed that “*the bare load cell and the clothespin prototypes were generally liked by clinicians, but they were also considered “cold” objects, so children are not genuinely interested in them*”. This necessitated the addition of emotional content, such as LEGO characters or colourful paintings on the crocodile toy, in this case, to increase attractiveness and intrinsic motivation. Moreover, Faraci et al. [49] reported their need to improve both the toys’ attractiveness and their solidity because the balls had broken 50% of the time during usage. Concerning ergonomics, Mironcika et al. [23] stated that the physical design of their tokens was constrained by the sensor size, leading to tokens that were perceived by therapists as possibly too large and uncomfortable for one-hand manipulation Additionally, another more pressing challenge is to address the lack of personalisation by developing mechanisms that can offer tailored learning experiences that adapt to the user’s performance and learning patterns [37].

The results of this review show the relationship between the intended use of smart objects and the electronic components employed in their realisation. Inertial (i.e., accelerometers, gyroscopes, or IMUs) and force sensors (i.e., FSR, load cells, or strain gauges) are mainly used in motor assessment and treatment, as they enable accurate quantification of kinematic parameters necessary to evaluate, monitor, or recognise motor performance and activity. Smart objects developed for cognitive assessment, treatment, and education often include, along with inertial sensors, optical, tactile, or tag sensors, as well as displays and LEDs. These electronic devices enable the detection of object proximity, gestures, and touch interactions, as well as the visualisation of images or geometric shapes, allowing for the use of such smart objects in cognitive games such as memory, shape matching, and assembly tasks. The features captured during these tasks provide detailed behavioural patterns that can be associated with specific cognitive processes. In addition, the use of custom sensors allows for more flexibility in the design process. For instance, Stephens-Fripp et al. [30] stated that using sensors fabricated with conductive ink would allow for greater control over the device’s size and shape and potentially yield more accurate results than commercial FSRs. Moreover, Bobin et al. [31] modified the shape of commercial sensors to conform the jack’s rounded design, resulting in a minimum detection threshold. Additionally, an important limitation concerns the use of sensors with large physical dimensions and low sampling rates. Such characteristics constrain the design of smart objects, often resulting in objects with increased size and reduced ease of handling during interaction. Moreover, low sampling rates limit the achievable performance in tasks requiring temporal resolution, such as gesture recognition [49]. A potential solution would be the integration of smaller and higher-performance sensors. However, this approach typically implies higher costs.

Other limitations identified in the reviewed studies relate to the hardware power consumption. The use of Wi-Fi communication or a large number of embedded electronic components, such as LED, vibration motor, and FSR, requires high energy, which leads to a significant reduction in battery life, limiting the device’s autonomy. These problems are particularly relevant for smart objects designed for daily activities or long-term use because they would require more frequently battery replacement or recharging [35,40,44,45,58,59].

### 4.2. Intended Use

From the analysis of the intended use of smart objects, it emerges that the most common smart objects are those designed for assessment and treatment purposes. This prevalence reflects the strong interest in developing tools capable of measuring cognitive and motor performance. However, the categorisation adopted in this review should be interpreted as a description of the main intended use reported in each study, rather than as the definition of smart objects. In practice, several systems show the potential to support multiple purposes beyond their original design. For example, Patel et al. [38] employed smart objects to train a machine learning-based classification method. However, the same combination of sensorised objects and classification algorithms could also be used for early assessment of neurodevelopmental disorders, thus extending their intended use to cognitive assessment. Similarly, smart objects with toy or daily life shapes, especially when equipped with visual and auditory feedback, may also serve educational purposes, such as teaching daily activities, colours, or basic cognitive skills. More generally, objects developed for assessment can be employed in treatment scenarios by providing feedback or guiding users through exercises, while objects designed for rehabilitation may generate data suitable for performance evaluation or for training machine learning models. This multifunctionality highlights the versatility of smart objects, which can be adapted to different contexts and application scenarios depending on how they are used. As a result, smart objects should be considered flexible tools whose intended use can evolve over time across clinical, educational, and research settings.

### 4.3. Experimental Session

The findings regarding experimental evaluation reveal a field in transition from development to actual implementation and evaluation with target users. Although most of the studies (67%) evaluated smart objects with target participants, they are still in an exploratory phase, with a focus on verifying feasibility, usability, and technical functionality before moving towards real-world applications. This initial validation allows researchers to identify the limitations and advantages of using smart objects in real-world settings, enabling them to improve these objects in terms of hardware, software, and design.

Although smart objects were conceived for use across the lifespan, from childhood to older adulthood, research has predominantly focused on children and adolescents, resulting in limited exploration of adult populations, which should be more systematically addressed in future studies. For example, future research could investigate the digitalisation of tests using smart objects to stimulate cognitive and physical functions in adults with neurodegenerative diseases, such as Parkinson’s disease or Alzheimer’s disease.

Furthermore, most of the articles reported only pilot studies involving a limited number of participants. These pilot studies enable researchers to investigate the feasibility and usability of the developed smart objects, which means that the results may not be sufficient to assess participants’ performance or draw robust statistical inferences. Moreover, almost half of the smart objects included in this review were tested on healthy children or adults, even when the devices were intended for patients with neurological or motor impairments. Consequently, this makes it difficult to compare data collected from smart objects with results obtained from standardised tests, such as the MABC-2 and the Wechsler Adult Intelligence Scale (WAIS) [21,23,25]. Additionally, the lack of clinical trials limits the ecological validity and practical relevance of these findings, and there is limited evidence regarding the usability of these smart objects in real-world contexts or their acceptance by clinicians and patients. The use of highly structured experimental tasks may also make it difficult to generalise the results to such settings. For example, some tasks did not reflect real daily activities or natural behaviours, as in studies [1,54], where participants simulated a tea-making task, or in study [45] in which object was tested in a controlled environment.

### 4.4. Data Integration, Management, and Processing

Regarding software components, several reviewed studies identify the integration of machine learning techniques as a key direction for future development [29,31,35,43,44,51,52]. In contrast, other works applied machine learning models but trained them using data collected from non-target populations, such as healthy adults, even when the intended application concerned children or patient groups [1,22,36,38,39]. This mismatch between training data and target users may limit the generalisability and reliability of the resulting models. More broadly, the integration of sensor-derived data with interpretable AI approaches could support the identification of relevant user-specific patterns and enable more personalised feedback or intervention strategies. Recent work in health management has shown how interpretable machine learning models can translate individual data into personalised intervention guidance [60]. However, these models need large amounts of data and high computational power. For these reasons, smart objects still rely on offline or cloud-based data processing, which requires data transfer and limits the possibility of real-time feedback. In future developments, they could be integrated with neuromorphic technologies [61,62] and/or edge sensors [63]. These two approaches may allow data to be processed directly on the smart object, transmitting only high-level features and enabling real-time feedback. Moreover, they could reduce power consumption and computational costs, addressing the limitations of traditional data processing.

Another challenge concerns data visualisation, as therapists need to create a dedicated tool that meets their specific requirements, identifies the most important information to display, and assesses the overall usefulness of the collected data [40]. Finally, the smart objects should be integrated within an environment that includes other devices to create a more robust and versatile ecosystem for user. Additionally, smart objects have to face interoperability limitations. For example, Faraci et al. [49] were unable to simultaneously connect all the nine sensor nodes, and Stephens-Fripp et al.’s [30] design has yet to be integrated with other sensors.

### 4.5. Limitations

We acknowledge that this scoping review has some limitations. First, the search strategy was designed to cover the multidisciplinary nature of smart objects by including Scopus, Web of Science, and PubMed. Nevertheless, relevant studies may have been missed because the search was limited to these three databases, to publications written in English, and to a predefined set of terms used to describe smart objects and related technologies. This aspect is particularly relevant in this field, where similar systems may be described using different terminology across engineering, healthcare, human–computer interaction, and education. In addition, studies whose full text could not be retrieved were not included, which may have further reduced the coverage of the available literature.

## 5. Conclusions

This review provides a structured overview of smart objects that are conceived and applied beyond traditional interaction paradigms. Specifically, it focuses on smart objects developed for clinical and educational contexts, intended either as tools for the assessment and treatment of cognitive and motor skills or as instruments for collecting data to support the development of machine learning models. Through a systematic analysis of the literature, this work examines the design characteristics of smart objects, classifying them according to their physical form. In addition, the review analyses the embedded electronic components and the parameters measured during human–object interaction, highlighting the sensing and feedback elements adopted in their development, and these are adapted to specific intended applications.

In conclusion, the research on smart objects for clinical and educational contexts reveals a field in transition, showing a great potential for treatment, assessment, and education. However, although some studies have involved patients, they are still early prototypes, constrained by technological limitations and by the absence of robust validation in real clinical or educational environments. Future research should aim to develop smart objects as robust, adaptable, and intelligent tools capable of operating beyond controlled experimental settings. Achieving this goal will require combining modular and user-centred design with reliable sensing, low-power operation, and data-processing strategies that can support long-term and natural interaction. At the same time, larger datasets collected directly from representative target users will be essential to develop reliable and personalised machine learning models and to adapt object behaviour, feedback, and activities to individual needs. These technological developments should progress alongside the involvement of clinicians, therapists, and other relevant stakeholders to ensure usability, acceptance, and meaningful interpretation of the collected data. Finally, the integration of emerging technologies, such as smart materials and neuromorphic processing, could further enhance the adaptability of smart objects. Neuromorphic approaches could support real-time processing of interaction data, while smart materials could enable objects to dynamically modify physical properties, such as shape or stiffness, thereby providing immediate and responsive feedback to the user. These advancements could support the creation of adaptable user activities by enhancing user engagement and promoting sustained interaction over time. In the future, these objects could also allow patients to perform activities at home without the presence of a clinician that can monitor them at a distance.

## Figures and Tables

**Table 2:** Barriers, challenges, and future research topics.

Topic	Barrier/Limitation	Challenge/Opportunity	Research Topic
Design and Hardware Selection	Object-specific design	Use different shapes of the smart object, improving the modularity and scalability.	Modular design
			Personalised experience with the smart object
	Attractiveness	The object is not interesting for users to ensure long-term use or integration in daily life.	Improve the attractiveness of the object
	Shape, size, and ergonomics [23,35]	The physical form is not adequate for patients with motor deficits.	Object shapes suited to target users
		Generate new type of haptic feedback, such as temperature, roughness, and changing dimension.	Smart material
	Commercial sensors	Control over the device’s size and shape could provide more accurate results.	Custom sensors
		Sensors with large dimensions or/and slow sampling rates [49].	Integrate small or/and high-performance sensors
	Fragile materials	The material is very fragile and breaks very easily [49].	Improve the solidity of material
	Power consumption [58,59]	Limit the autonomy of device.	Integrate sleep mode and use low-power sensors
Experimental Session	Limited number of participants	Design experimental sessions involving a larger set of participants.	Enlarge the number of participants
	The majority of the papers involve healthy participants	Target participants with cognitive or physical function loss were not included.	Recruit participants that have cognitive and physical constraints
	Lack of clinical trial	No evidence regarding their usability in real-world contexts.	Manage the ethical and security issues for testing in clinical settings
		Also involve the clinical stakeholder.	Verify the acceptance by clinicians and patients
	Structured tests [1]	Acquire data from a protocol with strict rules that could not be easily applied in real-world use.	Investigate natural interaction with the object
	Confounding factors during testing	Caregivers or researchers sometimes influence interactions, affecting the validity of the data.	Mitigate observer bias in interaction datasets
	Few papers involve older adults	Explore the use of smart objects with older adults.	Digitalise tests that stimulate the physical and cognitive functions of older adults
Data Integration, Management, and Processing	Aggregate and visualise collected data [40]	Create a dedicated data visualisation tool that meets their specific expectation.	Co-design with relevant stakeholders
	Manage a large amount of data	Small computing capabilities embedded on the smart object.	Investigate neuromorphic technology or edge computing strategy
	Integrate the smart object into a complex sensorised environment [30,49]	Interoperability between system components.	Functional ecosystem
	Machine learning limitations	ML models are trained on small datasets, usually adults or healthy subjects.	Create a large dataset from target users

## Data Availability

No new data were created or analysed in this study. Data sharing is not applicable to this article.

## Supplementary Materials

The following supporting information can be downloaded at: https://www.mdpi.com/article/10.3390/s26185812/s1, Table S1: Overview of smart objects included in the review, classified by physical shape and embedded electronic components. Black bullets mean the corresponding component is embedded in the object; Table S2: Characteristics of experimental validation for prototypes and tested smart objects.
