# Supplementary material for: Smart Objects for Clinical Rehabilitation and Education: A Scoping Review of Design, Sensing, and Validation
Source: Sensors (Basel). 2026 Sep 14;26(18):5812. doi: 10.3390/s26185812 (PMC13611113; doi:10.3390/s26185812)
Supplement: Supplementary file 1 [file sensors-26-05812-s001.zip › sensors-4456412-supplementary.pdf]

Review

Smart Objects for Clinical Rehabilitation and Education: A Scoping Review of Design, Sensing, and Validation

Lorenzo Pugi, Laura Fiorini *, Marco Vincenzo Maselli and Filippo Cavallo

Department of Industrial Engineering, University of Florence, 50121 Firenze, Italy; lorenzo.pugi@unifi.it (L.P.); marcovincenzo.maselli@unifi.it (M.V.M.); filippo.cavallo@unifi.it (F.C.)

* Correspondence: laura.fiorini@unifi.it

Table S1 Overview of smart objects included in the review, classified by physical shape and embedded electronic components. Black bullets mean the corresponding component is embedded in the object.

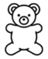 Toy shape, 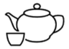 Daily life objects, 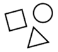 Geometrical shape, 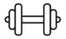 Other objects, 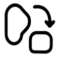 Cross-object Design

Reference	Shape	Inertial sensor	Optical sensor	Force sensor	Physiological sensor	Tag id	Tactile sensor	Environment sensor	Visual feedback	Audio feedback	Haptic feedback	External PC	Microcontroller
(Ammi et al., 2018)	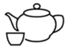	●		●		●		●	●				●
(Baber et al., 2017, 2019)	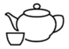	●		●					●	●	●		●
(Bobin et al., 2019)	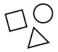	●		●									●
(Borghese et al., 2019)	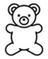			●			●						●
(Brons et al., 2021)	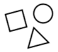	●							●				●
(Cano et al., 2020)	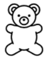	●				●			●				●
(Chandankhede et al., 2024)	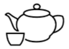	●		●				●	●	●			●

(Cholitha Hettiarachchi et al., 2022)	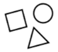	●	●						●		●		●
(Chubinidze et al., 2024)	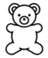	●		●			●				●		●
(Crovari et al., 2019)	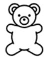	●				●	●		●	●			●
(Dujić Rodić et al., 2023)	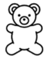			●					●	●			●
(Beccaluva et al., 2022)	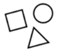	●				●			●	●			●
(Jovanov et al., 2016)	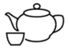	●			●		●						●
(Faraci et al., 2018)	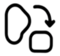	●						●					●
(Gutiérrez García et al., 2017)	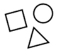	●	●					●	●	●			●
(Karime, Mahfujur Rahman, et al., 2011)	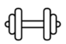	●		●							●		●
(Karime, Rahman, et al., 2011)	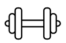	●									●		●
(Lanini et al., 2019)	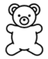	●											●
(Lee et al., 2016)	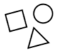	●	●										●
(Leo et al., 2022)	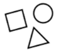	●					●					●	
(Lunardini et al., 2021)	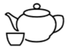	●		●					●				●
(Martim et al., 2018)	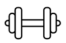	●		●									●

(Meriggi et al., 2021)	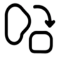	●										●	
(Mironcika et al., 2018)	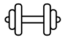	●							●				●
(Moraiti et al., 2015)	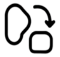			●									●
(Peng et al., 2021)	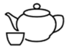	●		●									●
(Ramesha et al., 2022)	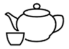 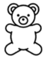	●											●
(Stephens-Fripp et al., 2019)	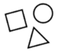			●									●
(Udayagiri et al., 2024)	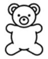		●									●	
(Varesano & Vernero, 2012)	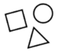	●	●					●			●		●
(Vonach et al., 2016)	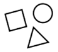				●					●			●
(Wang et al., 2018)	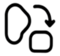	●											●
(Westeyn et al., 2012)	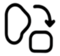	●					●						●
(Martinho et al., 2024)	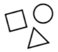			●									●
(Patel et al., 2024)	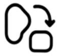	●										●	
(Bartolomei et al., 2025)	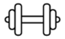	●							●	●			●
(Solorzano Alcivar et al., 2025)	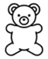								●	●			●

(Vondrak et al., 2025)	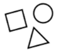		●						●	●			●
(Mohandas et al., 2025)	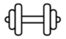	●	●						●	●			●
(Pinos Cisneros et al., 2026)	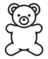	●							●	●	●		●
(Biswas et al., 2026)	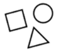	●				●			●	●	●		●

Table S2 Characteristics of experimental validation for prototypes and tested smart objects

Reference	Target population	Number of participants	Age	Health status of participants	Function of the object	Experimental Setting	Experimental evaluation
(Ammi et al., 2018)	Non-target-user evaluation	15	N/A	Neurotypical	Home monitoring in daily activities in post stroke rehabilitation	Laboratory corridor	Technical / bench; Reliability/accuracy;
(Baber et al., 2019)	Non-target-user evaluation	20	Young adults	Neurotypical	Animate objects to support the cueing of action sequences	The School of Electronic, Electrical and Systems Engineering	Reliability / accuracy
(Baber et al., 2017)	Non-target-user evaluation	23	Young adults	Neurotypical	Animate objects as part of coaching in activities of daily living	The School of Electronic, Electrical and Systems Engineering	Reliability / accuracy
(Bobin et al., 2019)	Target-user evaluation	3 in preliminary test<br>9 in the preliminary study	Young adults, Middle-aged adults, Older adults	Post-stroke	Monitor motor functions (fingers' orientation and pressures, and tremors) of stroke patients during rehabilitation sessions	Rehabilitation center in Le Havre.	Technical / bench; Usability/acceptability; Reliability/accuracy;
(Borghese et al., 2019)	Target-user evaluation	N/A	Children	N/A	Hand rehabilitation for young patients	N/A	Technical / bench; Usability/acceptability; Reliability/accuracy; Reference-standard comparison

(Brons et al., 2021)	Target-user evaluation	95	Children	49 children with motor development problems and 46 healthy children	Evaluate the fine motor skill level of children	Elementary school	Reliability/accuracy; Reference-standard comparison
(Cano et al., 2020)	Target-user evaluation	N/A	Children	children with cochlear implants and children whose communication is sign language	Involve the STEM methodology for children with special needs	N/A	Usability/acceptability
(Chandankhede et al., 2024)	Target-user evaluation	17	N/A	Post-stroke	Home monitoring of stroke patients' arm and hand motor activity	N/A	Technical / bench; Reliability/accuracy
(Cholitha Hettiarachchi et al., 2022)	Target-user evaluation	27	Children	Neurotypical	Develop the cognitive skills of children and help them to learn better while playing and exploring the physical word	N/A	Usability/acceptability

(Chubinidze et al., 2024)	Target-user evaluation	26	Adolescent, Young adults, Middle-aged adults	People with eating disorders	Emotion Regulation therapy	3 ED centers	Usability/acceptability; Intervention study; long-term evaluation
(Crovari et al., 2019)	Target-user evaluation	5	Adolescent, Young adults	People with neurodevelopmental disorders (NDD)	Engage people with NDD in variety of customised tasks	N/A	Usability/acceptability
(Dujić Rodić et al., 2023)	Target-user evaluation	14	Children	Neurotypical	Teach young children about geometric shapes	Controlled laboratory setting	Technical / bench; Usability/acceptability; Reliability/accuracy
(Beccaluva et al., 2022)	Target-user evaluation	12	Children	Children with intellectual disability	Train memory skills by mean of games with the cubes	Daycare center	Usability/acceptability;

(Jovanov et al., 2016)	Non-target-user evaluation	N/A	N/A	N/A	Monitor usage of the bottle	N/A	Technical / bench
(Faraci et al., 2018)	Target-user evaluation	10	Children	Neurotypical	Anticipate the diagnosis of autism spectrum disorders, neuro developmental disorders and social fragilities	N/A	Technical / bench; Reliability/accuracy
(Gutiérrez García et al., 2017)	Target-user evaluation	65	Children	N/A	Enhance decision-making process during children's psychomotor delay screenings	Public nursery school	Reliability/accuracy; Reference-standard comparison
(Karime, Mahfujur Rahman, et al., 2011)	Non-target-user evaluation	15	N/A	Neurotypical	Rehabilitation of upper limbs for people with wrist injuries	Discover laboratory, University of Ottawa	Usability/acceptability

(Karime, Rahman, et al., 2011)	Non-target-user evaluation	7	Young adults, Middle-aged	Neurotypical	Rehabilitation of upper limbs for people with wrist injuries	Discover laboratory, University of Ottawa	Technical / bench; Usability/acceptability
(Lanini et al., 2019)	Target-user evaluation	2	Children	Children with autism	Anticipate the diagnosis of autism spectrum disorders	Department of "Neuroscienze dell'età evolutiva" at Stella Maris institute	Technical / bench; Reliability/accuracy; long-term evaluation
(Lee et al., 2016)	Target-user evaluation	86	Young adults	Neurotypical	Assess fine-motor proficiency, visual-motor integration, low-level working memory, attention span and cognitive problem solving	N/A	Reliability/accuracy; Reference-standard comparison
(Leo et al., 2022)	Target-user evaluation	20	Young adults	Neurotypical	Investigate how spatial skills influence performances in a haptic task	N/A	Reliability / accuracy; Reference-standard comparison

(Lunardini et al., 2021)	Target-user evaluation	43	Middle-aged adults, Older adults	Neurotypical	Detect age-related changes in writing and tremor parameters during daily-life handwriting	N/A	Technical / bench; Reliability/accuracy; Reference-standard comparison
(Martim et al., 2018)	Non-target-user evaluation	N/A	N/A	Neurotypical	Monitor the evolution of the gait and balance during physical rehabilitation process	N/A	Technical / bench
(Meriggi et al., 2021)	Target-user evaluation	10	Children, Adolescent	People with cerebral palsy	Innovative paediatric rehabilitation for children with unilateral cerebral palsy	CARE lab	Usability/acceptability; Reference-standard comparison; Intervention study
(Mironcika et al., 2018)	Target-user evaluation	30	Children	Children with motor development problems	Assess fine motor skills in children	Primary Schools in Amsterdam	Usability/acceptability; Reference-standard comparison

(Moraiti et al., 2015)	Target-user evaluation	N/A	N/A	N/A	Assess motor skills	Healthcare institutions and school	Usability/acceptability; long-term evaluation
(Peng et al., 2021)	Target-user evaluation	26	Young adults	Neurotypical	Human activity recognition and identification of subjects	N/A	Technical / bench; Reliability/accuracy
(Ramesha et al., 2022)	Target-user evaluation	10	Children	Children with autism	Identify children's motor skills and detect atypical children in early stage	N/A	Usability / acceptability; Reliability / accuracy
(Stephens-Fripp et al., 2019)	Non-target-user evaluation	1	Middle-aged	Neurotypical	Drive sensory feedback mechanisms and record the impact of this feedback for prosthesis hands	N/A	Technical / bench; Reliability/accuracy; Reference-standard comparison

(Udayagiri et al., 2024)	Non-target-user evaluation	15	Adults	Neurotypical	Assessment of neurodevelopmental impairments in the first two years of age	N/A	Technical / bench; Reliability/accuracy
(Varesano & Vernero, 2012)	Target-user evaluation	5	Older adults	Neurotypical	Device for video games and leisure activities	University of Tyrin, local library. Training centre class.	Usability/acceptability
(Vonach et al., 2016)	Target-user evaluation	8	Children	Neurotypical	Measure children’s physiological parameters to assess their health	Participant's homes	Technical / bench; Usability/acceptability; Reference-standard comparison
(Wang et al., 2018)	Target-user evaluation	52	Children	Children’s post trauma stress	Assess the children's post trauma stress	Kindergartens of children	Reference-standard comparison; Reliability / accuracy

(Westeyn et al., 2012)	Target-user evaluation	5 children in preliminary free-play		Neurotypical	Early identification of children with autism	N/A	Technical / bench; Usability/acceptability; Reliability/accuracy
(Martinho et al., 2024)	Non-target-user evaluation	N/A	N/A	N/A	Address current shortcomings of post-stroke patient rehabilitation assessment	N/A	Technical / bench; Reliability/accuracy
(Patel et al., 2024)	Non-target-user evaluation	10	Young adults	Neurotypical	Assessment of manual exploratory behaviours	Due to COVID, the experiment was conducted remotely	Technical / bench; Reliability/accuracy
(Bartolomei et al., 2025)	Non-target-user evaluation	6	Adults	Neurotypical	Support children with neurodevelopmental disorders in enhancing social and emotional interactions during therapy sessions	ISTC-CNR	Technical / bench; Reliability/accuracy

(Solorzano Alcivar et al., 2025)	Target-user evaluation	35	Children	N/A	Improve cognitive and motor skills in toddlers aged two to four year	Child Development Centers	Usability/acceptability
(Vondrak et al., 2025)	Non-target-user evaluation	N/A	N/A	N/A	Support neurocognitive recovery stimulating memory, attention, and executive function	N/A	Technical / bench; Reliability/accuracy
(Mohandas et al., 2025)	Non-target-user evaluation	N/A	N/A	N/A	Improve the engagement of children who have difficulties engaging with the world around them	N/A	Design / prototype description; no smart-object evaluation identified
(Pinos Cisneros et al., 2026)	Target-user evaluation	5	Children	People with cerebral palsy	Motivate hand therapy at home	Home	Usability/acceptability; Intervention study; long-term evaluation
(Biswas et al., 2026)	Target-user evaluation	N/A	Children, Adolescent	N/A	Monitoring, characterising and enhancing human spatial reasoning	School	Technical / bench
